# Detection of occult hepatitis B in serum and oral fluid samples

**DOI:** 10.1590/0074-02760170071

**Published:** 2018-01

**Authors:** Moyra Machado Portilho, Leticia Cancella Nabuco, Cristiane Alves Villela-Nogueira, Carlos Eduardo Brandão-Mello, José Henrique Pilotto, Geane Lopes Flores, Lia Laura Lewis-Ximenez, Elisabeth Lampe, Livia Melo Villar

**Affiliations:** 1 Fundação Oswaldo Cruz-Fiocruz, Instituto Oswaldo Cruz, Laboratório de Hepatites Virais, Rio de Janeiro, RJ, Brasil; 2 Universidade Federal do Rio de Janeiro, Hospital Universitário Clementino Fraga Filho, Rio de Janeiro, RJ, Brasil; 3 Universidade Federal do Estado do Rio de Janeiro, Hospital Universitário Gaffrée e Guinle, Rio de Janeiro, RJ, Brasil; 4 Hospital Geral de Nova Iguaçu, Nova Iguaçu, RJ, Brasil; 5 Fundação Oswaldo Cruz-Fiocruz, Instituto Oswaldo Cruz, Laboratório de AIDS e Imunologia Molecular, Rio de Janeiro, RJ, Brasil

**Keywords:** occult hepatitis B, HBV DNA, oral fluid

## Abstract

In occult hepatitis B infection (OBI), hepatitis B virus DNA (HBV DNA) can be detected in serum samples; however, oral fluid collection for detection of HBV DNA has not yet been explored, despite the availability of collection devices. Serum and oral fluid samples from 45 hepatitis B core antibody (anti-HBc)-positive patients were collected for the amplification of the HBV polymerase gene. HBV DNA was detected in five serum and four oral fluid samples (the detection limit for oral fluid was 1.656 log IU/mL in paired serum). In conclusion, simple methodologies of sample collection and in-house polymerase chain reaction (PCR) allowed detection of HBV DNA, and these could be used to improve the diagnosis of OBI, especially in locations with limited resources.

Occult hepatitis B (OBI) is identified by the presence of hepatitis B virus DNA (HBV DNA) in the liver (with detectable or undetectable HBV DNA in the serum) of individuals deemed HBV surface antigen (HBsAg)negative, using currently available assays. When detectable, the amount of HBV DNA in the serum is usually very low (< 200 IU/mL). Patients can be divided into two groups: seropositive-OBI [hepatitis B core antibody (anti-HBc)-and/or hepatitis B surface antibody (anti-HBs)-positive] and seronegative-OBI (anti-HBc-and anti-HBs-negative) (Raimondo et al. 2008).

The persistence of HBV DNA in HBsAg-negative individuals could be related to the poor laboratory detection of HBsAg owing to the low levels of HBs antigenemia or HBsAg mutants, underlying coinfections, or other host-related factors (Bréchot et al. 2001). It has been demonstrated that patients who are intravenous drug users or have other diseases that are also transmitted parenterally, such as hepatitis C and HIV, are at a greater risk of developing occult hepatitis B (Morsica et al. 2009), and are more likely to develop complications such as cirrhosis (Squadrito et al. 2013).

Although HBV DNA is always present in liver tissue, its clinical assessment is difficult (Larrubia 2011); therefore, the use of sensitive methods for HBV DNA detection in serum samples is paramount. In contrast, oral fluid samples have been used in molecular studies to detect hepatitis B (Van der Ejik et al. 2005, Kidd-Ljunggren et al. 2006, Heiberg et al. 2010) owing to the ease by which these samples can be obtained in subjects with difficult venous access, more particularly children, the elderly, drug addicts, and patients on haemodialysis. Furthermore, oral fluid sample collection is cheaper, less invasive, and painless compared to blood collection, and can easily be performed in remote laboratory environments. Therefore, we investigated HBV DNA detection in oral fluid samples from individuals with occult hepatitis B.

This study aimed to detect OBI in a cohort of anti-HBc- and/or anti-HBs-positive patients using serum and oral fluid samples.

A total of 45 individuals were included in this study; however, all of them did not present HBsAg in serum. These individuals were recruited from the following ambulatories in the region of Rio de Janeiro in the period from October 2010 to July 2014: Viral Hepatitis Ambulatory (IOC/Fiocruz), Clementino Fraga Filho Hospital (Federal University of Rio de Janeiro/UFRJ), Gaffree and Guinle University Hospital (Federal University of Rio de Janeiro State), and Nova Iguaçu General Hospital. Individuals provided informed consents prior to blood and oral fluid sample collection, and the study was approved by the Fiocruz ethics committee (CAAE 18281313.4.0000.5248). Individuals could be of any gender, race, or ethnicity, and had to be over 18 years of age.

Blood samples were collected by venipuncture, and the obtained serum was stored at -20°C until further use. Oral fluid samples were obtained using a Salivette collector (Sarstedt, Nümbrecht, Germany). This collection device consists of a polypropylene tube that contains an absorbent pad made from cotton. The swab was placed inside each volunteer's mouth for 2 min to absorb oral fluid. Salivettes were checked visually for blood contamination and excluded if such event had occurred. After collection, 1 mL of phosphate buffer saline (PBS, pH 7.2) was added to minimise the effects of oral fluid degradation and facilitate pipetting. The vials were centrifuged at 2000 x g for 10 min and stored at -20°C until use.

The serum samples were subjected to commercial enzyme immunoassays (ELISA) for the detection of HBsAg, anti-HBc, anti-HBc IgM, anti-HBs (Bioelisa anti-HBs, Biokit, Barcelona, Catalonia, Spain), hepatitis B e antigen (HBeAg), and anti-HBe (e411 Cobas, Roche Diagnostics, Manheim, Germany) according to the instructions of each manufacturer. Serum samples were also analysed to determine anti-HCV (Murex anti-HCV 4.0, DiaSorin, Kyalami, Republic of South Africa) and anti-HIV (DS-EIA-HIV-AGAB-SCREEN, RPC, Diagnostic System, Nizhny Novgorod, Russia) levels, as well as the biochemical dosages of liver enzymes, such as aspartate aminotransferase (AST), alanine aminotransferase (ALT), alkaline phosphatase, total, direct, and indirect bilirubin, and gamma-glutamyl transferase (GGT), using a commercial kit (LabMax 560, LabTest, Lagoa Santa, Brazil).

HBV DNA was extracted from serum samples using a commercial kit (High Pure Viral Nucleic Acid Kit, Roche Diagnostics, Mannhein, Germany) by following the manufacturer's instructions. To extract HBV DNA from oral fluid samples, a RTP® DNA/RNA Virus Mini Kit (Stratec Biomedical Ag, Berlin, Germany) was used. The oral fluid volume was increased two-fold (400 µL), as determined previously (Portilho et al. 2012), and the manufacturer's recommendations were followed.

Oligonucleotides were used to amplify the polymerase gene region of HBV (Mallory et al. 2011) *via* a single round of amplification, generating a product containing approximately 940 base pairs. For serum samples, PCR analysis was performed using a tube filled with 25 μL of reaction buffer containing the following components: 0.5 µM of each oligonucleotide, 0.2 mM of a mixture of four deoxynucleotides, 10× PCR buffer and 1.5 mM MgCl_2_, Platinum Taq polymerase (Invitrogen, San Diego, CA, United States) (5U) at 1.5 U, and target DNA (5 µL). A target-free control reaction tube contained 25 µL of reaction mixture only. Negative and Positive HBV controls were included for each target tested. The thermocycler (T3 Thermocycler, Biometra, Göttingen, Germany) program incubated the samples for 3 min at 95°C, followed by 45 cycles consisting of 95°C for 10 s, 58°C for 20 s, and 72°C for 40 s, followed by an additional extension step at 72°C for 5 min. To increase the PCR sensitivity in oral fluid samples, the protocol was modified as follows: 0.5 µL (2.5 U) of 5 U/µL Platinum Taq DNA polymerase, (Invitrogen) and 10 µL DNA.

Serum samples in which HBV DNA was detected using in-house PCR were also submitted for the quantification of HBV DNA *via* Abbott Real Time HBV (Abbott Laboratories), and for viral sequencing employing the same oligonucleotides as the ones used for PCR amplification (Mallory et al. 2011), to determine HBV genotypes. Sequences were analysed using the Mega v6.0 software (Tamura et al. 2013), and HBV genotypes were identified using blast alignment.

All individuals completed a questionnaire, and a descriptive statistical analysis was performed, with the means, medians, and standard deviations being calculated. Statistical analysis was performed using the Graph-Pad InStat software (La Jolla, CA, United States).

Most of the patients were men (29/45), and the mean age was 36.36 ± 20.74 years. All individuals were HBsAg-negative, anti-HBc-positive, and anti-HBs-negative. We could not access HBeAg or anti-HBe results for all patients. Among them, 30 were HBeAg-negative, and 12 out of 33 samples were anti-HBe-positive. Among the 45 individuals, 16 had detectable levels of anti-HCV, and 24 were anti-HIV-positive. Eleven patients were receiving treatment for hepatitis C and/or HIV infections during the study. Regarding the biochemical tests, the mean ALT value was 14.21 ± 13.18 U/L, and the mean AST value was 21.33 ± 26.49 U/L. The mean total bilirubin value was 0.15 ± 0.15 U/L, the mean alkaline phosphatase value was 65.17 ± 37.91 U/L, and the mean GGT value was 94.27 ± 120.95 U/L.

All serum samples were subjected to the in-house PCR protocol for HBV polymerase gene determination. Among them, five (11.11%) showed the presence of HBV DNA, displaying a mean viral load of 2.246 ± 0.635 log IU/mL. Genotypes were determined *via* sequence analysis in three of the five samples, in which two were classified as genotype F and one as genotype A. In two samples, the quality of the sequence data was very poor, which prevented their correct classification into genotypes. Among the patients with HBV DNA detected through the qualitative method in both types of sample, one was anti-HCV positive, and three were anti-HIV positive (Table).

**TABLE:** Demographic, serologic and biochemical details of serum hepatitis B virus DNA (HBV DNA) positive samples

Sample	Gender	Age	ALT/AST (U/L)	Anti-HIV	Anti-HCV	HBeAg	Anti-HBe	HBV DNA in serum (log IU/mL)	Qualitative HBV DNA in serum	Qualitative HBV DNA in oral fluid	HBV genotype
1	M	42	*	*	Non-reagent	*	*	1.623	Detected	Non detected	F
2	M	30	6/9	Reagent	Non-reagent	Non-reagent	Reagent	1.656	Detected	Detected	F
3	F	25	4/10	Reagent	Non-reagent	*	*	3.290	Detected	Detected	*
4	F	66	*	Reagent	Reagent	*	*	2.500	Detected	Detected	*
5	F	52	10/12	Non-reagent	Non-reagent	Non-reagent	Reagent	2.260	Detected	Detected	A

ALT: alanine aminotransferase; AST: aspartate aminotransferase; HBeAg: hepatitis B e antigen; HCV: hepatitis C virus;

* data unavailable.

Oral fluid samples, for which HBV DNA had been detected in paired serum samples, were also subjected to in-house PCR, and four of these contained HBV DNA. The oral fluid sample that was HBV-DNA-negative presented a viral load of 1.623 log IU/mL in its paired serum sample. The agarose gel electrophoresis results obtained for oral fluid samples are represented in Figure.

**Figure:**
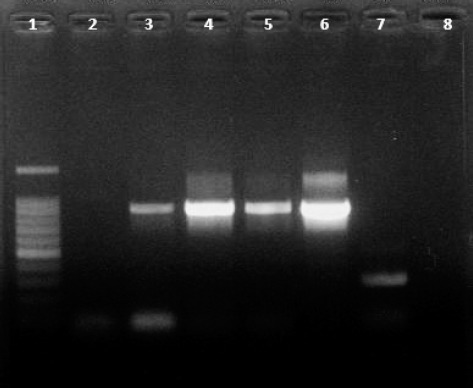
Oral fluid samples from individuals with occult hepatitis B infections. Lane 1: 100 bp molecular weight standard. Lanes 2-5: polymerase chain reaction (PCR) products of oral fluid samples. Lane 6: serum sample as positive control. Lane 7: serum sample as negative control. Lane 8: PCR Mix control without DNA template.

The presence of OBI was evaluated *via* detection of HBV DNA in serum and oral fluid samples in a cohort of patients with no HBsAg but showing anti-HBc and/or anti-HBs positive results. In the studied population (n = 45), five cases of OBI were confirmed where patients with no HBsAg presented HBV DNA in serum.

We found that one OBI patient was anti-HCV positive, and three were anti-HIV positive. Some studies indicate that OBI infection is more common in patients who are coinfected with hepatitis C or HIV, varying from 1-62% in HIV patients (Piroth et al. 2008), and occurring in approximately one-third of subjects from the Mediterranean Basin and in more than 50% of East Asian populations (Coppola et al. 2015). The presence of occult HBV in coinfected HCV patients may indicate more severe liver damage, cirrhosis, and increased rates of hepatocellular carcinoma (Chen et al. 2016). In HIV patients, the identification of OBI cases may be due to HBV immune-escape, which reduces the humoral immune response and anti-HBs titres, recurrence of HBV replication, recovery of immune responses after HIV treatment, or the development of resistance to lamivudine therapy (Maldonado-Rodríguez et al. 2015).

One of the difficulties in identifying OBI is the low level of HBV DNA in serum samples. Therefore, it is extremely important to use a sensitive PCR protocol. We evaluated the applicability of an in-house PCR method for amplification of the polymerase gene of HBV, which was able to detect a positive result in a sample containing 1.623 log IU/mL HBV DNA following the commercial method, even allowing the detection of different HBV genotypes, and presenting 100% of concordance between them.

To the best of our knowledge, oral fluid samples have not yet been evaluated for use in occult hepatitis B diagnosis. It is well-known that the viral load in oral fluid is proportional to that in serum (Zhang et al. 2008), and is higher in HBsAg- and HBeAg-positive individuals (Noppornpanth et al. 2000). Therefore, it is considered extremely difficult to detect HBV DNA in oral fluid samples from OBI patients. In this study, it was possible to detect HBV DNA in oral fluid of four of the five positive serum samples (80%).

HBV DNA could not be detected in only one oral fluid sample. This sample presented a low viral load (1.623 log IU/mL) in the paired serum sample. Non-detection may have been the result of viral load fluctuation, the DNA extraction method, or the collection device used. The extraction method (RTP® DNA/RNA Virus Mini kit, Stratec) and Salivette collector were used in a previous study in our laboratory (Portilho et al. 2012) to detect serial dilutions of HBV DNA in artificially infected samples. In this case, it was possible to detect 2 x 10^1^ copies of HBV DNA/mL. However, another study showed that collectors based on the principle of mechanical friction may be more efficient (Heiberg et al. 2010) for HBV DNA detection.

However, this study presents some limitations. First, we did not include OBI seronegative patients because of the large number of individuals already included in our laboratory cohort. Moreover, we did not have sufficient volumes of oral fluid samples to perform HBV DNA sequencing, and compare the detected genotypes using this type of sample. We intend to collect samples from patients with the serological profile of OBI to identify more cases. Furthermore, we could not conduct any additional sample collections from these individuals during the course of the infection because of logistical problems, and therefore, we could not determine the potential occurrence of viral load fluctuation in the oral fluid samples showing negative results.

In conclusion, it was possible to detect HBV DNA in serum and oral fluid samples from individuals with occult hepatitis B infection, suggesting the usefulness of this method and its ability to improve the identification of these cases in patients with difficult venous access.
